# All-optical, an ultra-thin endoscopic photoacoustic sensor using multi-mode fiber

**DOI:** 10.1038/s41598-020-66076-9

**Published:** 2020-06-04

**Authors:** Nadav Shabairou, Benjamin Lengenfelder, Martin Hohmann, Florian Klämpfl, Michael Schmidt, Zeev Zalevsky

**Affiliations:** 1 Faculty of Engineering, Bar-Ilan University, Ramat-Gan, 52900 Israel; 2 Institute of Photonic Technologies (LPT), Friedrich-Alexander-Universität Erlangen-Nürnberg (FAU), Konrad-Zuse-Straße 3/5, 91052 Erlangen, Germany; 3 Erlangen Graduate School in Advanced Optical Technologies (SAOT), Paul-Gordan-Straße 6, 91052 Erlangen, Germany

**Keywords:** Engineering, Optics and photonics

## Abstract

Photoacoustic endoscopy (PAE) is a method of *in-vivo* imaging that uses tissue absorption properties. In PAE, the main tools used to detect the acoustic signal are mechanical ultrasound transducers, which require direct contact and which are difficult to miniaturize. All-optic photoacoustic sensors can challenge this issue as they can provide contact-free sensing. Here, we demonstrate sensing of photo-acoustic signals through a multimode fiber (MMF) which can provide an ultra-thin endoscopic photoacoustic sensor. Furthermore, we show the advantage of using the optical-flow method for speckle sensing and extract the photoacoustic signal despite the mode-mixing along the MMF. Moreover, it is demonstrated for the first time that the speckle reconstruction method can be used without the need for imaging of the speckles as this enables the use of multimode fibers for the speckle method.

## Introduction

The Photoacoustic (PA) effect is a physical phenomenon which converts absorbed optical energy into acoustic energy. The physical mechanism of this effect relies on the absorption of a short pulse of light, leading to thermal expansion of an absorber which causes propagation of a mechanical wave^1^. Photoacoustic tomography (PAT) forms images by detecting the induced acoustic waves created by the PA effect^2^. In biological tissues, PAT has demonstrated a combination of high imaging contrast sensitivity to optical absorption and high spatial resolution at depth as a result of low acoustic scattering in soft tissue^3–5^. In the past decades, PAT has been rapidly developed and is applied to a wide range of biomedical applications: brain lesion detection^6^, hemodynamics monitoring^7^, and breast cancer diagnosis^8^. The detection of the acoustic signals, created by the PA effect, is mostly performed using ultrasound transducers^9–12^. This method requires direct contact and impedance matching with the sample in order to avoid acoustic reflections and signal losses. In clinical applications (such as burn diagnostics^13^, laser^14^, and brain surgery^15^) physical contact, coupling or immersion is undesirable or impractical. Optical ultrasound sensors offer an alternative to ultrasound transducers and have the ability to overcome this challenge. Remote, optical ultrasound sensors are therefore an attractive alternative to contactless transducers^16–19^. This is especially true for application in photo-acoustic endoscopy (PAE)^20,21^ since these systems require precise miniaturization. The optical ultrasound sensors are divided into two groups^22^: refractometry and interferometry. Non-contact methods^23^ based on refractometry detection are using the changes in the refractive index. The refractive index is changing according to mechanical pressure created by the PA signal. These methods require high ultrasound pressures which limits the potential for PAT. Interferometric methods^17,24,25^ show a higher sensitivity of PA signal detection. These methods are sensing the changes in the interference pattern of the reflected light caused by the vibrations of the tissue. However, interferometric methods are limited by inefficient light-collection from rough tissue–air interfaces, leading to low detection sensitivity^26^.

Optical coherence tomography (OCT)^27,28^ is an imaging method that uses low-coherence interferometry to produce a two-dimensional image of optical scattering from internal tissue microstructures. This method achieves a high spatial resolution. On the other hand, the OCT image has a lower contrast compared to PAT and suffers from sensitivity to speckle, polarization changes, and scattering losses. Moreover, the OCT penetration depth is limited to less than 2 mm for soft tissue, while PA can reach more than 1 cm. The combination of the two methods in one all-optic system was demonstrated and achieved by combining the OCT system with acoustic optical interferometry methods, for example adding Fabry Perot interferometry^29^.

Remote speckle sensing (RSS) is a method to analyze the vibration of a rough object^30^. If a coherent light source, such as a laser beam, illuminates a rough surface, then the reflected beam light will accumulate a random phase. This causes the self-interference of the beam and creates a random speckle pattern. This speckle pattern is directly affected by the surface roughness and it will shift according to the surface tilt. By analyzing these speckle shifts the acoustic vibration of the object can be measured from a great distance without the need for a physical coupling. The use of remote speckle sensing to detect a PA signal has already been shown^19,26^. However, it requires a direct line of sight between the sensor and the object which limits the use in clinical and intra-body applications. In order to provide a flexible solution that makes this method applicable for intra-body applications, the use of an optical fiber for speckle pattern transmission is necessary. Multimode fibers (MMFs) are optical fibers that show the ability of image transmission^31–34^ and can be used as a suitable option for our task. In MMF, the information is transmitted using a variety of modes that exist in the fiber. It allows for the transmission of large amounts of information, while at the same time its size remains relatively small^35,36^. This feature is a source of interest for telecommunication^37^ and endoscopy applications^38–40^. However, local defects or bending along the fiber length cause differences in the ray’s travel path and create transitions between the modes. This phenomenon is called modal scrambling^41^ and causes an ‘unpredictable’ output beam profile. Due to this modal scrambling, the correlated speckle shift is translated into a chaotic motion. Although image reconstruction through MMF is possible with prior knowledge of the state of the fiber and the modal scrambling, in remote speckle sensing, an imprecise reconstruction of the pattern, with even very small error, can have a huge effect on the sensing results. For this reason, it’s essential to have a method that enables tracking the speckle pattern change without the need for image reconstruction.

In this work, we use the Farnebäck^42^ optical flow algorithm to overcome this problem and demonstrate, for the first time to our knowledge, optical speckle sensing without the need for imaging or image reconstruction. We had achieved a high SNR for PA signal detection through MMF. This has been done with images captured from the distal end of the MMF without any preprocessing, prior knowledge of the state of the fiber and the mode mixing. This combination of MMF and remote speckle analysis for the aim of intra-body PAE is unique and has not been shown before. This method is robust, has high sensitivity and can be used as an ultra-thin optical transducer. Therefore, the potential for an all-optical PAE is demonstrated.

## Results and Discussion

### Photoacoustic measurements

**Figure 1:**
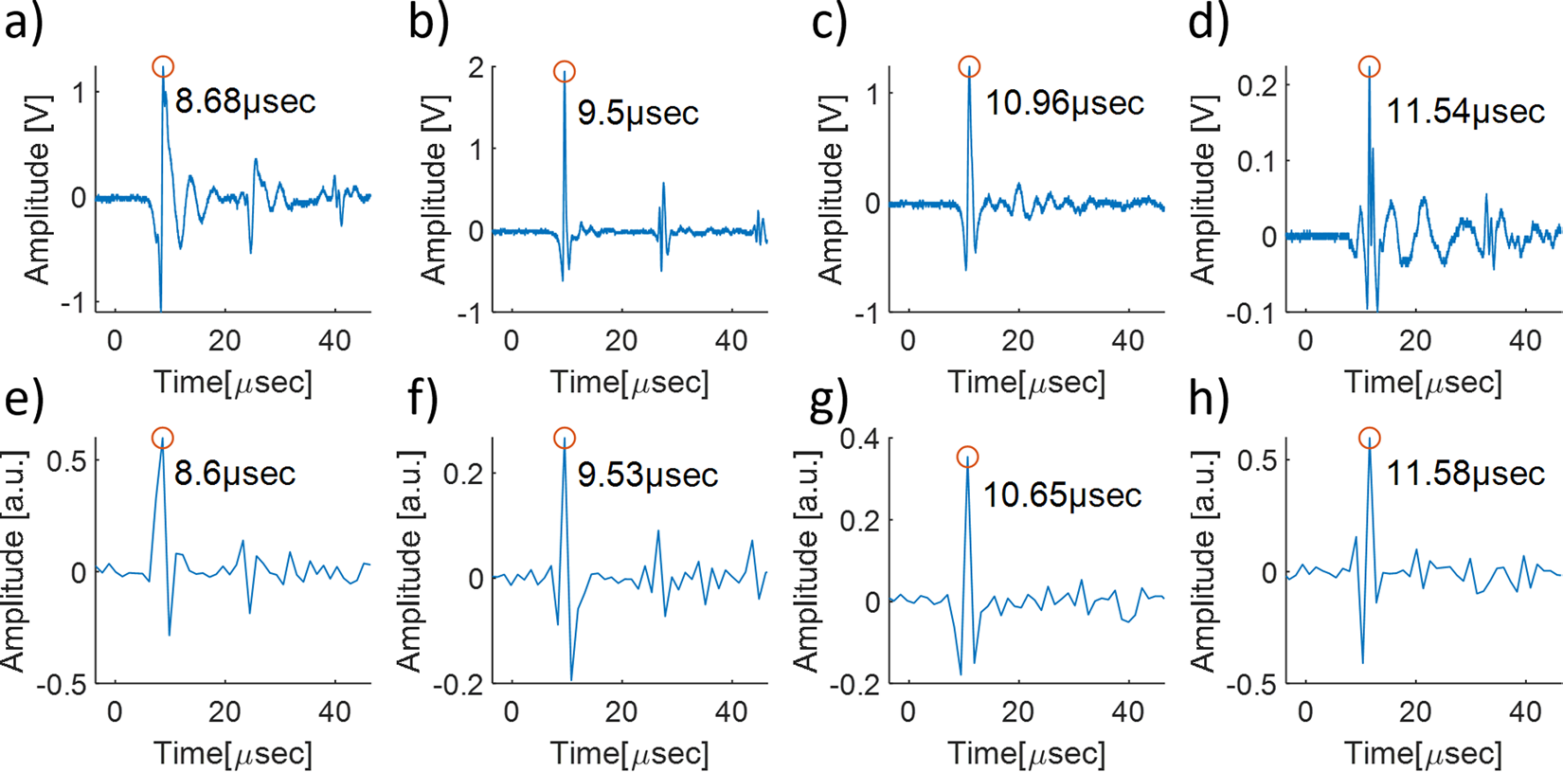
Comparison between sample surfaces vibration measurements using ultrasound transducer (UST) (**a–d**) and multimode fiber remote speckle sensing (MMF-RSS) (**e,f**). Negative time points are related to measurements before the photoacoustic excitation.

In this experiment, we conducted a comparison between measurements with a multimode fiber and ultrasound transducer (UST). Figure 1 shows the comparison results for the three PVCP samples with different thicknesses and the ex-vivo sample. The measurements of the PA signal with UST are shown at the upper row (Fig. 1(a–d)), compared to the MMF-RSS measurements at the lower row (Fig. 1(e–h)). The highest peak in the figures is the time when the acoustic signal reaches the surface of the phantom for the first time. In parts of the measurements, it is possible to see the reflected signal resulting from the reflection of the initial signal back and forth from the anterior surface. The time between the reflected signal and the initial signal peaks was approximately twice the time required for the initial signal to reach the anterior surface.

For sample 1, the initial peak was detected by the UST at 8.68 μs compared to 8.6 μs measured by MMF-RSS. The times between the reflected and the initial signal peaks were 16.48 μs and 15.85 μs in Fig. 1(a,e) respectively which was approximately twice the time of the initial peak with minor inaccuracy caused by the limitation of temporal resolution. For sample 2, the initial peak was detected by UST at 9.5 μs compared to 9.53 μs measured by MMF-RSS. The times between the reflected and initial signal peaks were 18.06 μs and 17.06 μs in Fig. 1(b,f) respectively. Figure 1(c,g) show the measurements with sample 3 which was thicker than samples 1 and 2. The initial peak was detected by the UST at 10.96 μs compared to 10.65 μs measured by MMF-RSS, whereas the reflected signal was detected neither by UST nor by MMF-RSS. For the ex-vivo sample, the initial peak was detected by the UST at 11.54 μs compared to 11.58 μs measured by MMF-RSS. Another noticeable observation was that similarly to UST measurements, the reflected signal was weaker than the initial signal in the MMF-RSS measurements. This indicates the ability of the method to distinguish between the different signal amplitudes which is needed for the realization of a future photoacoustic imaging system.

The signal to noise ratio (SNR) was calculated according to $20{\log }_{10}\left(\frac{{A}_{peak}}{\sigma }\right)$, ${A}_{peak}$ is the initial peak value and *σ* is the standard deviation of the signal before the trigger. The SNR mean value for MMF-RSS measurements was 28 dB compared to 39 dB measured by the UST. The PA spectrum was between 100kHz-2MHz while the center frequency of 200kHz-600kHz allows measuring the PA signal with the high-speed camera for the proof of concept study in this work.

**Figure 2:**
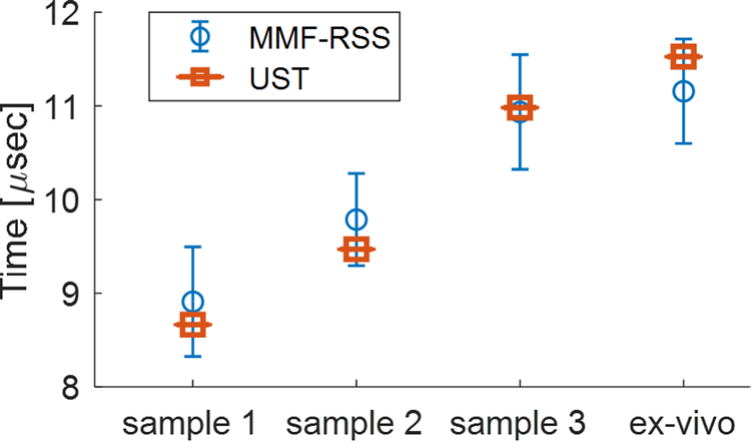
A statistical analysis of the initial peak measured by MMF-RSS and UST for four different samples, the blue circle represents the mean value of measurements made with MMF-RSS and the blue bars its standard deviation (N = 15). The orange square represents the corresponding measurements made with the ultrasonic transducer.

Figure 2 shows the mean value of the time at which all the initial peaks were measured by MMF-RSS and UST for four different samples. In the figure, the blue circle represents the mean value of measurements made with MMF-RSS and the blue bars its standard deviation (N = 15). For sample 1 through 3, the values are as follows: 8.9 μs ± 0.6 μs, 9.8 μs ± 0.5 μs, 10.9 μs ± 0.6 μs, respectively. For the ex-vivo tissue sample, the signal was detected at 11.2 ± 0.5 μs. The orange square represents the corresponding measurements made with an ultrasonic transducer which are 8.7 μs, 9.5 μs, 11.0 μs and for samples 1 through 3 respectively. The PA initial peak measured by UST for the ex-vivo tissue sample appears at 11.5 μs. The measurements done by MMF-RSS are consistent with the transducer and show a clear match between the detection times of the transducer and the time intervals defined by the MMF-RSS. The standard deviation for the MMF-RSS detection is expected at a range of ±0.6 μs and can be explained as a result of the camera sampling rate which leads a time window of 1.2 μs between the frames. This time window is limiting the axial resolution to $\Delta x=1.6\,mm$. A camera with a faster sampling rate will reduce the size of the time window that will lead to a smaller standard deviation and higher axial resolution. The lateral resolution is defined by the Illumination spot size.

By calculating the distance between the initial peak and the reflected peak, it is possible to determine the speed of sound in the material. Table 1 shows the speed of sound in the material using the relative time between the initial and reflected signal. The speed of sound was calculated by dividing the thickness of the phantom in half the time between peaks. For PVCP phantom, the average speed of sound is $1349\,\frac{{\boldsymbol{m}}}{{\boldsymbol{s}}}$ which corresponds to previous measurements done by an ultrasound thickness measurement device. The speed of sound measured in the ex-vivo sample is $1424\,\frac{{\boldsymbol{m}}}{{\boldsymbol{s}}}$, consistent with measurements in the literature^43^.

**Table 1:** Calculation of the speed of sound in the material using the relative time between the initial and reflected signal, from N measurements.

Phantom	Thickness $({\boldsymbol{mm}})$	N	Time Between Peaks (µ sec)	Speed Of Sound $(\frac{{\boldsymbol{m}}}{{\boldsymbol{s}}})$
Sample 1	10.2	14	14.97	1364
Sample 2	11.5	11	16.95	1360
Sample 3	13.6	10	20.60	1323
Ex-vivo	13.4	11	18.84	1424

### System sensitivity measurements

**Figure 3:**
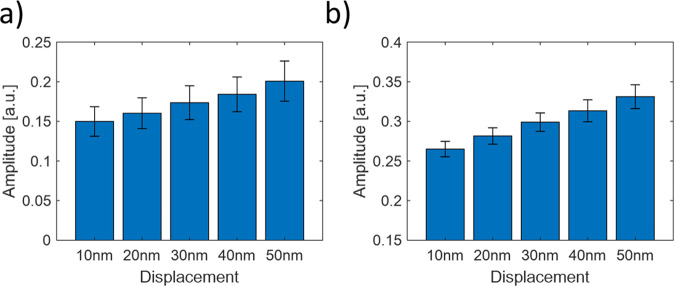
Displacement sensitivity done with 128 × 16 pixels frame size (**a**) and with 400 × 400 pixels (**b**). The black bars are the standard deviation (N = 50).

The system sensitivity was measured by moving the sample with a piezoelectric positioner (611.3 S Nanocube, Physik Instrumente). This is intended to simulate the surface displacement caused by the PA wave. The sample was moving with a constant jump vertically to the sample’s surface (Supplementary Fig. 1). Figure 3 shows the results of the system measurements for the displacement size of 10 nm, 20 nm, 30 nm, 40 nm, and 50 nm. Figure 3(a,b) show the measurements done with 128 × 16 pixels and with 400 × 400 pixels (full fiber core image) respectively. The results show the system has a displacement sensitivity of less than 20 nm. When using 128×16 pixels, the displacement sensitivity is reduced to 20 nm. For the full-frame, the sensitivity reaches 10 nm. The more speckles are present, the better is the displacement sensitivity. From the minimum detectable displacements ${u}_{min}$, we can find the minimum detectable pressure ${p}_{min}$ by^18^:

1 $${p}_{min}=\pi Z{u}_{min}\,f$$

Thereby, *Z* is the acoustic impedance of the specimen which is about $1.4\times 106\,\frac{Pa\cdot s}{m}$ for our ex-vivo sample, and *f* is the ultrasonic frequency. For the ultrasonic frequency of 5 MHz, the minimum detectable pressure is 22 *kPa*. By comparison, the interferometry method allows approaches of less than 1 nm detectable displacements and ultrasonic frequency of 180 *Pa*^22^ in some ultrasonic frequency. This would require an improvement in our system sensitivity in the future.

### Preliminary imaging results

**Figure 4:**
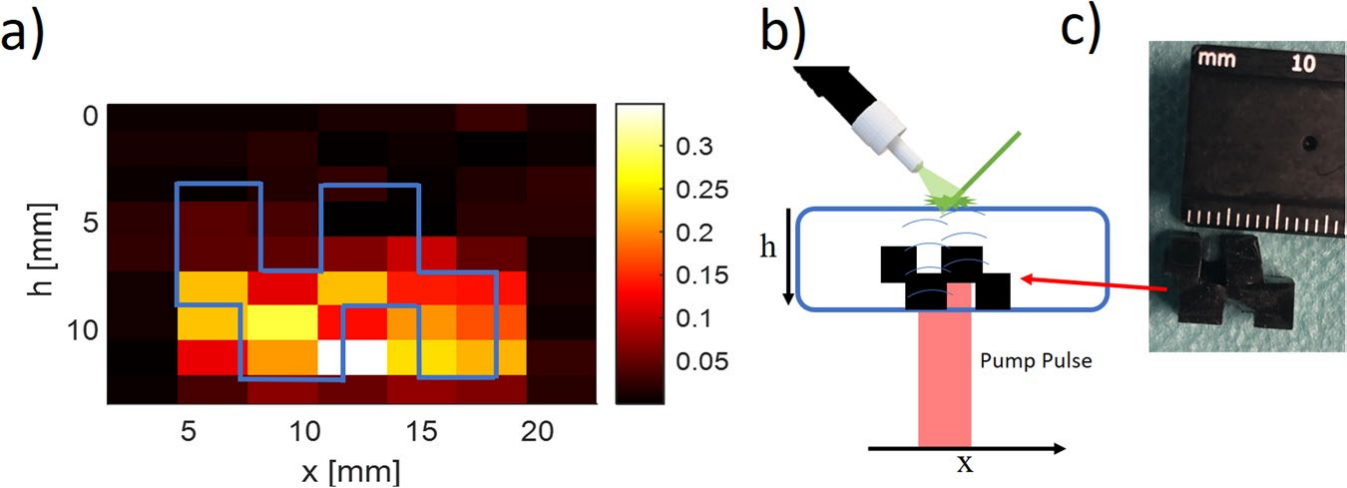
(**a**) A depth phantom imaging, the image done by moving the pump laser over the back of the phantom and measured the arrival time of the PA signal to the surface, the blue frame represented the absorption core scale and shape. (**b**) The absorption core with a two-step shape. (**c**) The Phantom cross-section.

Figure 4(a) shows the phantom imaging done by moving the pump laser over the back of the phantom. Unlike the previous phantoms, the current phantom contained an absorbent core with a two-step shape as seen in Fig. 4(b,c). Due to the fact that the excitation beam diameter was 3 mm, it simultaneously excited photoacoustic waves on two surfaces with different heights. The PA source locations were calculated by the arrival time of the signal multiplying the speed of sound in the material. In the center column of the image (Fig. 4(a)), the measured PA signal shows the excitation of two surfaces with different heights. This creates two signals with a time difference between them, depending on their distances. This causes the two peaks in the center column. The use of a wide beam detracts from the spatial resolution of the system. In contrast, a smaller and more focused beam will allow the spatial resolution of several microns. But the smaller the area of excitation, the shorter the PA signal and the higher the need for a faster measuring device. For this reason, our system is limited to a wide beam of light as the camera sampling rate was not high enough. Therefore, by using a camera or a faster measuring device, higher axial and lateral resolution can be achieved.

## Conclusions

Our work shows a proof-of-concept of using a single multi-mode fiber for a photo-acoustic contact-free optical transducer. We have shown the PA detection using all-optical detection while using phantoms that have similar mechanical and optical properties to those of soft tissues. As the main outcome, we have shown that an optical flow algorithm can be used in optical speckle sensing without the need for imaging. This allows us to achieve high sensitivity for the surface displacement despite modal scrambling in the MMF. Hence, the detectors can be easily reduced to a diameter of 100 microns and below at the distal end in the future.

The temporal resolution in this work was relatively limited due to the sampling speed of the camera and does not provide high-resolution PA measurements. But this issue is solvable, as already today high-speed cameras with a sampling rate of 10 Mfps are existed in the market^44^ and over time, the cameras will be faster and cheaper.

Additionally, MMF can serve as all in one solution which will provide optical resolution PAE (OR-PAE), and acoustic resolution PAE (AR-PAE). The optical and the acoustic resolution could be achieved by focusing the light through the MMF^45,46^, whether it’s focusing the excitation light to gain OR-PAE or focusing the illumination light to gain acoustic resolution. This system will have a size of less than a few hundreds of microns and can be operated inside very thin areas.

## Methods

### Sample preparation

**Figure 5:**
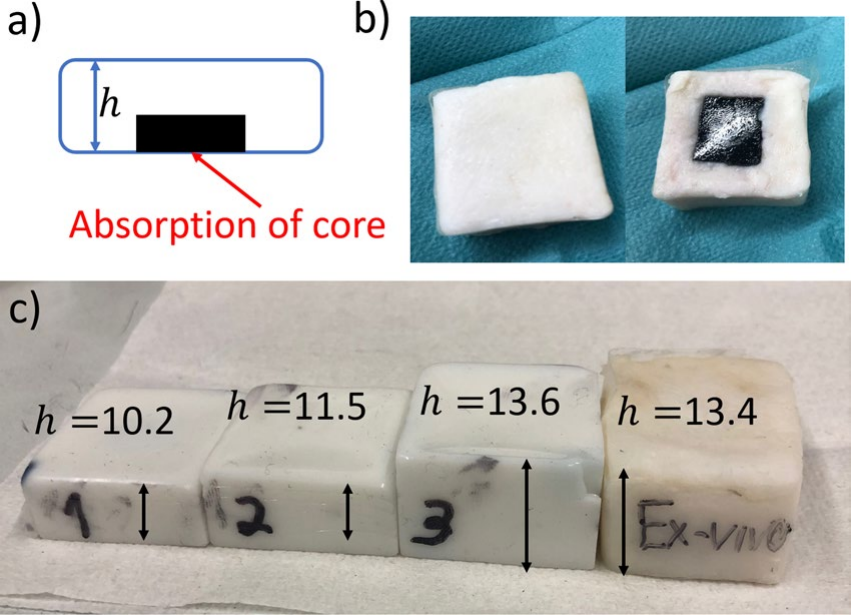
The PVCP phantoms used in this work consist of an absorbing black part with a surrounding scattering matrix. (**a**) Phantom cross-section *h* is the phantom thickness. (**b**) *Ex-vivo* sample front and back. (**c**) The three PCVP phantoms by numbering and the ex-vivo pork fat phantom.

Figure 5 shows details of the experimental samples that include a tissue phantom and an ex-vivo porcine fat tissue obtained from a local supermarket. Preparation of the samples was done according to the protocol of our previous work^19^, Polyvinylchloride plastisol (PVCP, Standard Lure flex (medium), Lure Factors, Great Britain) was used as phantom material since it offers long term stability and similar mechanical properties as soft tissue^47^. The speed of sound in the phantoms was measured using an ultrasound thickness measurement device (Mini-Test 430, Elektro Physik, Germany) connected to a piezoelectric sensor head with a resonance frequency at 2 MHz at 1330 $\frac{m}{s}$. The density *ρ* was measured by volume displacement of ethanol at $1040\,\frac{kg}{c{m}^{3}}$. The resulting acoustic impedance ($Z=\rho c$) of the used phantoms in this work is $1.38\times {10}^{6}\frac{kg}{{m}^{2}s}$ which is in good agreement with the values of soft tissue: The impedance of fat tissue is $1.4\times {10}^{6}\frac{kg}{{m}^{2}s}$ and for muscle $1.62\times {10}^{6}\frac{kg}{{m}^{2}s}$^48^.

In order to adjust the optical properties, additives were added during the preparation process. A black plastic color was added to change the absorption coefficient ${\mu }_{a}$ and $Ti{O}_{2}$-particles were added to adjust the reduced scattering coefficient ${\mu }_{s}^{{\prime} }$. In this work, a color-concentration of 7 Vol-% and a $Ti{O}_{2}$-concentration of $4\frac{mg}{ml(PVCP)}$ was used for the absorbing and scattering phantom parts. The optical properties for these concentrations were determined at the excitation wavelength of 1064 nm using spectrophotometric measurements and Inverse Adding Doubling. In this work, the absorption coefficient for the absorbing phantom part is 106 $\frac{1}{cm}$ and the reduced scattering coefficient for the scattering part is $21\frac{1}{cm}$ . The scattering coefficient for the absorbing part and the absorption coefficient for the scattering part can be neglected. For the experiments carried out in this work, three square PVCP-phantoms with a size of 3 cm × 3 cm are manufactured consisting of a scattering matrix and an absorbing core. The phantoms are produced in a two-step process. First, the absorbent core is manufactured so that each core has a size of 1 cm ×1 cm square and a thickness of 5 mm. Second, the absorbing target was put on the bottom and the cast was filled until the final phantom height $h$ is reached. The geometrical distances are measured using a caliper. The ex-vivo sample consists of an absorber placed inside porcine fat. A hole with the dimensions of absorbing target was cut out of the fat tissue and the absorber was placed inside this hole. A thin coating of ultrasound gel on the absorber ensures good acoustic coupling. The speed of sound for the fat tissue is assumed at $1450\,\frac{m}{s}$^43^.

### Experimental setup

**Figure 6:**
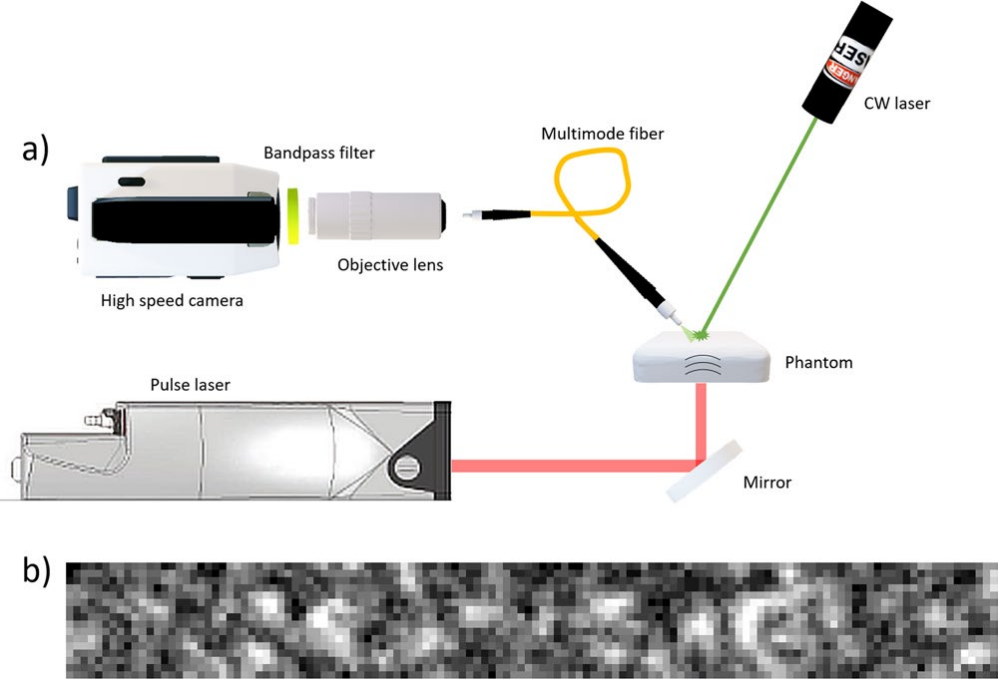
Speckle sensing with the multi-mode fiber setup: (**a**) A CW-laser is used for illumination of the phantom and for speckle generation. The speckles are transferred to the high-speed camera via the multi-mode fiber. (**b**) An exemplary speckle pattern image captured by the camera.

Figure 6 illustrates the experimental setup used for PA sensing with optical speckles through a multimode fiber. PA excitation of the phantom was done using a single short laser pulse (Quantel laser, Les Ulis (France), Q-Smart 450) with a wavelength of 1064 nm and a pulse duration of 5 ns. The laser pulse energy was 26.5 *mJ* with a beam diameter of 3 mm, leading to a maximum energy exposure of 93.7 $\frac{mJ}{c{m}^{2}}$. This exposure is below the maximum exposure limit for PA excitation for short laser pulses at 1064 nm on soft tissue (100 $\frac{mJ}{c{m}^{2}}$,^28^). The anterior surface of the phantom was illuminated with a CW-laser of 532 nm wavelength (which generates the speckle pattern), while the back of the phantom was hit by the excitation beam at the absorption tissue. A multimode optical fiber (105 µm core diameter, Thorlabs M15L02) was placed 4-6 mm from the anterior of the phantom and used for the collection of scattered light. At the end of the fiber, the light was magnified by an objective (100X Mitutoyo Plan Apo Infinity Corrected Long WD Objective, M=100, NA=0.7, working distance 6 mm) and imaged on the high-speed camera sensor (Phantom v1210, pixel size 28 μm, Vision Research, USA). The camera recorded with a sampling rate of 820,500 frames per second and an image resolution of 128 × 16 pixels. This sampling rate leads to a time window of 1.2 μs between the frames, which is too low for precise PA sensing. However, it is high enough for the proof of concept demonstration in this work. The camera acquisition was triggered by the excitation laser pulse. For verification of the PA measurements with MMF-RSS, we used a broadband contact UST (V109-RM, Olympus Corporation, Japan) with a resonance frequency of 5 MHz. Contact USTs are considered to be the state of the art for PA signal detection with high precision compared to the remote speckle sensing approach.

### Signal analysis

The captured video was analyzed with an optical-flow algorithm from MATLAB (R2018b, The MathWorks, Inc., Narick, MA, USA) toolbox. The algorithm estimates the direction and speed of a speckle’s grains from one video frame to another using the Farnebäck method^42^. This method is used with polynomial expansion transform to approximate the neighbors of a pixel. The method also combines Image Pyramids to detect large displacements and Gaussian filter to smooth out the neighboring displacements.

**Figure 7:**
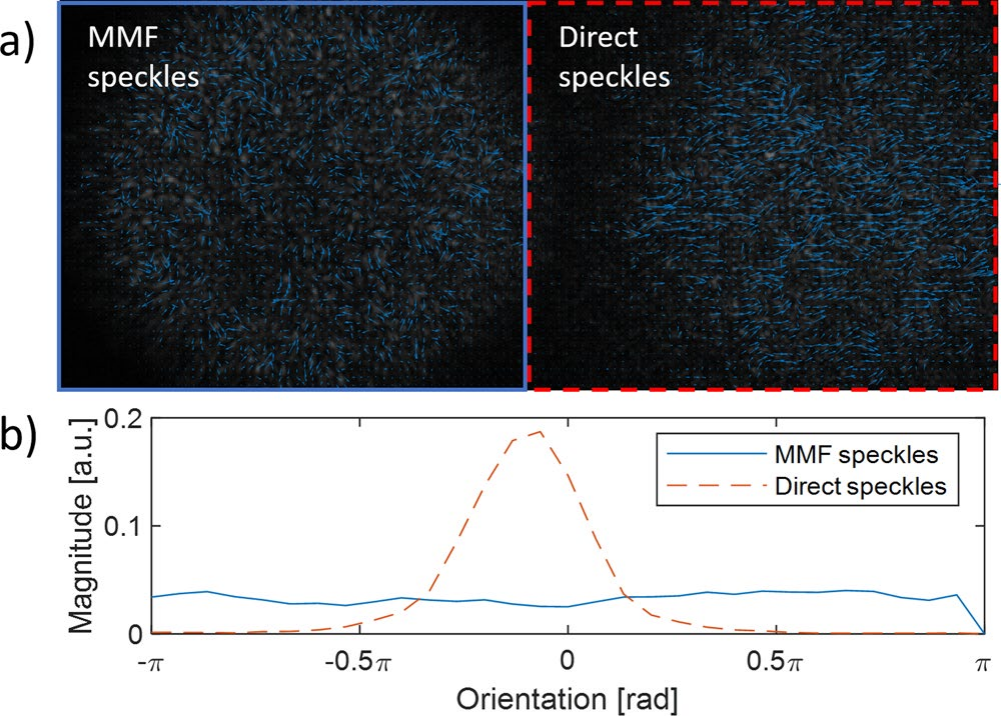
Comparison between MMF speckles shifts and direct speckles shifts (without MMF) created by surface vibrations. (**a**) the arrows represented the direction and speed of the speckles in the image. (**b**) The distribution of the overall velocities as a function of a velocities orientation.

Figure 7 is demonstrating the algorithm estimation for MMF speckles shifts and direct speckles shifts created by surface vibrations, with a view to show the differences created by the modal scrambling. The direct speckles are speckles that are received directly from the object surface and only pass through a lens (Supplementary Fig. 2), as it was done in secondary speckle remote sensing^30^. The MMF speckles are speckles that are received through a MMF, similar to the setup in Fig. 6. In Fig. 7(a) the arrows represented the direction and speed of the speckles in the image. The effect of the modal scrambling in MMF on the speckle shift can be seen in Fig. 7(b). While the direct speckles have common orientation caused by the direction of the surface tilt, this orientation is been lost through the MMF and translated to chaotic orientations. This fact limits the applicability of the peak correlation method which was used so far to analyze the vibration of the surface^19^.

In this work, the temporal signal was calculated according to Eq. (2):

2 $$Signal(t)=sign({V}_{x}(t)+{V}_{y}(t))\times (m(t)-\tilde{M})$$

When *m(t)* is the mean of magnitudes of the estimated velocities at time *t*, $\tilde{M}$ is the mean value of *m(t)* at the time before the trigger. The use of the mean value of each magnitude was chosen to give an indication of the general change in the speckle pattern, as it is not affected by shifting in different directions. *V*_*x*_ and *V*_*y*_ are the mean value of the velocity’s directions in Cartesian coordinate and *sign* is Sign function. $sign({V}_{x}(t)+{V}_{y}(t))$ intended to give the information about the overall movement direction in each frame. This part determines the sign of the signal while *m(t)* determines the magnitude of the signal amplitude.

## Supplementary information

Supplementary information.
