# Supplementary material for: All-optical, an ultra-thin endoscopic photoacoustic sensor using multi-mode fiber
Source: Sci Rep. 2020 Jun 4;10:9142. doi: 10.1038/s41598-020-66076-9 (PMC7272416; doi:10.1038/s41598-020-66076-9)
Supplement: Supplementary file 1 — Supplementary information. [file 41598_2020_66076_MOESM1_ESM.docx]

All-optical, an ultra-thin endoscopic photoacoustic sensor using multi-mode fiber

Nadav Shabairou^a,*^, Benjamin Lengenfelder^b,c^, Martin Hohmann^b,c^, Florian Klämpfl^b,c^ Michael Schmidt^b,c^, Zeev Zalevsky^a,c^

^a^ Faculty of Engineering, Bar-Ilan University, Ramat-Gan, Israel, 52900;

^b^ Institute of Photonic Technologies (LPT), Friedrich-Alexander-Universität Erlangen-Nürnberg (FAU), Konrad-Zuse-Straße 3/5, 91052 Erlangen, Germany

^c^ Erlangen Graduate School in Advanced Optical Technologies (SAOT), Paul-Gordan-Straße 6, 91052 Erlangen, Germany

* E-Mail: shabain@biu.ac.il

Supplementary Materials


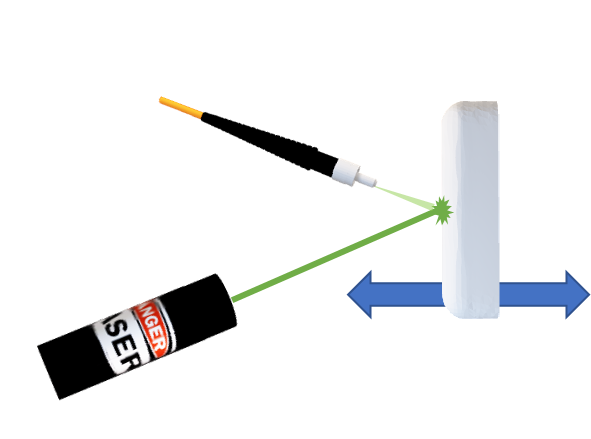


Figure 1: Displacement sensitivity setup, the sample was moved with a piezoelectric positioner on the vertical axis to its surface. This is intended to simulate the surface displacement caused by the photoacoustic wave. A CW-laser is used for illumination of the sample and for speckle generation The speckles transferred to a CMOS camera via the multimode fiber.


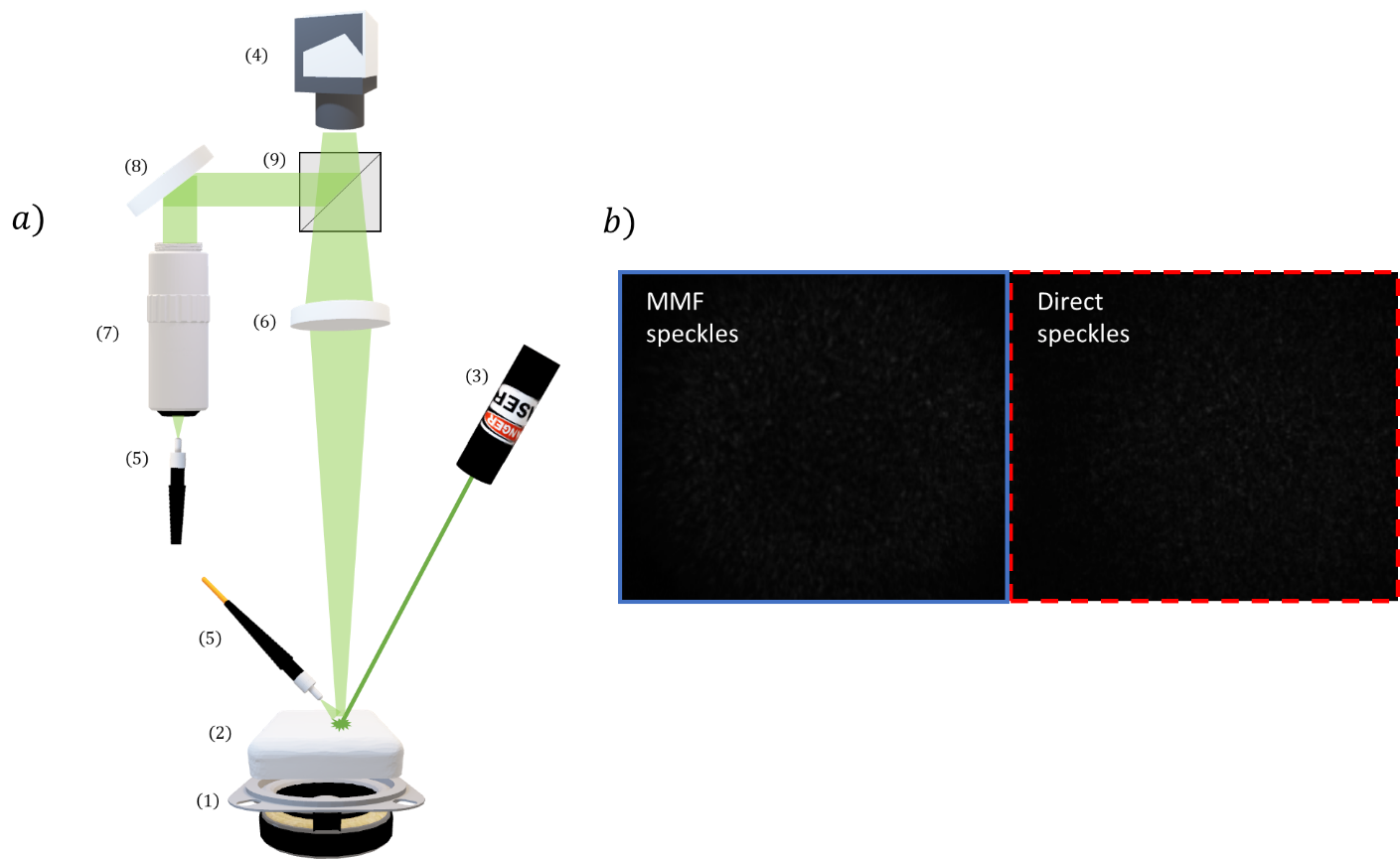


Figure 2: a) The setup used to compare between multimode fiber and direct speckles. A loudspeaker (1) is used to generate different oscillation frequencies which are coupled to the phantom (2). A CW-laser (3) is used for illumination of the phantom and for speckle generation. The speckles are transferred to a CMOS camera (4) in two paths: via the multimode fiber (5) and direct. The direct speckles are received directly from the object surface and only pass through a lens (6). The speckles which transmitted multimode fiber are magnified using 20x objective (7) and transmitted to the CMOS camera via a mirror (8) and beam-splitter (9). b) An exemplary speckle pattern image captured by the camera.
